# Systems approaches to understand oxygen sensing: how multi-omics has driven advances in understanding oxygen-based signalling

**DOI:** 10.1042/BCJ20210554

**Published:** 2022-02-04

**Authors:** Michael Batie, Niall S. Kenneth, Sonia Rocha

**Affiliations:** Institute of Systems, Molecular and Integrative Biology, University of Liverpool, Biosciences Building, Crown Street, Liverpool L697ZB, U.K.

**Keywords:** genomics, hypoxia, hypoxia inducible factors, proteomics, transcriptomics

## Abstract

Hypoxia is a common denominator in the pathophysiology of a variety of human disease states. Insight into how cells detect, and respond to low oxygen is crucial to understanding the role of hypoxia in disease. Central to the hypoxic response is rapid changes in the expression of genes essential to carry out a wide range of functions to adapt the cell/tissue to decreased oxygen availability. These changes in gene expression are co-ordinated by specialised transcription factors, changes to chromatin architecture and intricate balances between protein synthesis and destruction that together establish changes to the cellular proteome. In this article, we will discuss the advances of our understanding of the cellular oxygen sensing machinery achieved through the application of ‘omics-based experimental approaches.

## Introduction

Molecular oxygen is best known for its connection to oxidative phosphorylation and ATP production. Changes to oxygen availability give rise to complex cellular and organismic responses in all multicellular organisms. Hypoxia is defined as a condition in which oxygen demand exceeds supply. Physiological responses to hypoxia in humans and other mammals have been long appreciated and known [1]. However, how cells sense and response to hypoxia at the molecular level is still under investigation. A major advancement in this area occurred in the late 1990s and early 2000s with the identification of hypoxia inducible factors (HIFs) [2], a family of transcription factors regulated by changes to cellular oxygen levels [3–5]. This seminal work laid ground for additional studies delineating the hypoxia signalling pathway and its importance was noted by the award of the Nobel Prize in Physiology or Medicine 2019 to three investigators who led on these discoveries, Greg Semenza, Peter Ratcliffe and William Kaelin Jr [6].

## Hypoxia signalling

Following the discovery of HIF, intensive research efforts identified the main components of the hypoxia signalling pathway in cells. HIFs are heterodimers containing an oxygen-sensitive α subunit, of which there are three homologues, HIF-1α, HIF-2α and HIF-3α, and a constitutively expressed β subunit (HIF-1β, also referred to as the aryl hydrocarbon receptor nuclear translocator or ARNT) [7]. The mechanisms by which HIFs are controlled by oxygen revealed the existence of a class of enzymes, sensitive to molecular oxygen availability, named prolyl-hydroxylases (PHDs, gene names *egln1*, *egln2* and *egln3*). In well oxygenated cells and tissues prolyl hydroxylation of HIF-α subunits creates a high affinity binding site for the E3 ligase complex composed of Von Hippel–Lindau tumour suppressor (VHL), Elongin B/C and cullin 2 [8,9]. The VHL complex promotes the conjugation of Lysine 48 (K48)-linked ubiquitin chains to HIF-α subunits leading to their proteasomal degradation. PHDs require 2-oxoglutarate, iron and molecular oxygen to efficiently promote this modification and are part of the 2-oxoglutarate dependent dioxygenase (2-OGDD) superfamily [9]. Another dioxygenase, belonging to this class of enzymes, factor inhibiting HIF (FIH), promotes primarily asparagine hydroxylation [10]. This modification located in the transactivation domain of HIF-α and prevents binding to the co-activator p300/CREB binding protein (CBP), an interaction important for full activation of HIF-dependent target genes [10]. More recently, and based on structural, biochemical and bioinformatic analysis, more enzymes have been identified as belonging to the 2-OGDD class [9,11,12]. These enzymes have roles in controlling chromatin structure, transcription, mRNA fate and processing, translation and protein stability [13]. It thus became apparent that the research should move into these domains of biology, as well as try to understand the role of HIFs in these processes.

**Figure 1.**
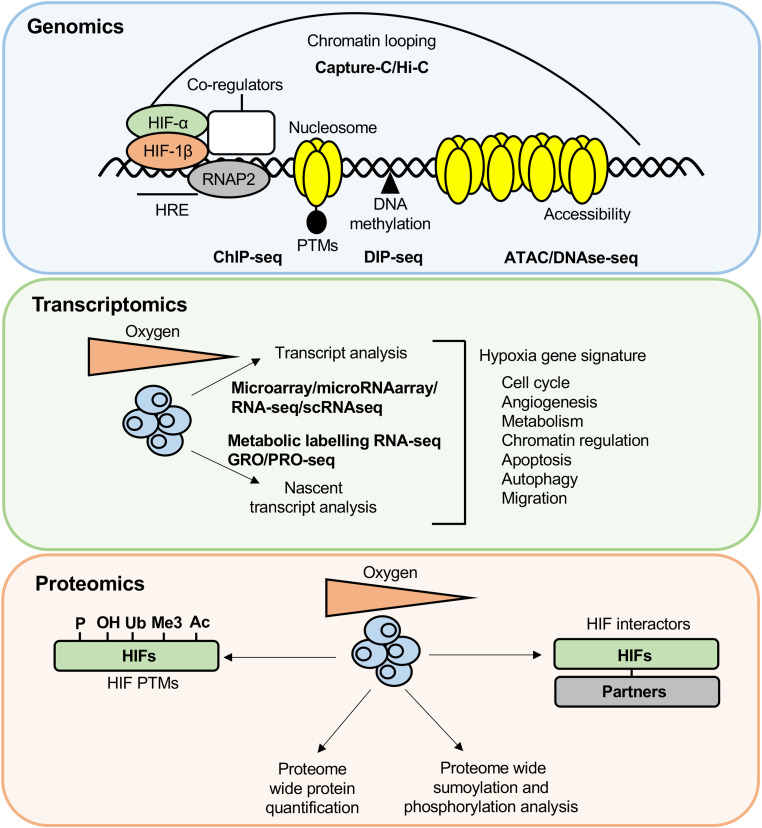
Genomics, transcriptomics and proteomics approaches used to study hypoxia. *Genomics*: ChIP-seq has measured genome wide histone PTM changes in response to hypoxia and has also defined pan genomic HIF binding sites. DIP-seq has measured genome wide changes DNA methylation in response to reduced oxygen. ATAC-seq and DNase-seq have analysed changes in chromatin accessibility in response to hypoxia. The chromatin confirmation capture techniques, Capture-C and Hi–C have mapped chromatin looping interactions in response to hypoxia. *Transcriptomics*: A variety of transcriptome profiling technologies have been used to determine transcriptome responses to hypoxia, which are primarily mediated by HIF, and reveal a hypoxic gene signature. *Proteomics*: Mass spectrometry approaches have elucidated proteome wide changes in protein levels in response to hypoxia and sumoylation and phosphorylation changes across the proteome. Mass spectrometry techniques have also identified HIF subunit binding partners and post-translational modifications of HIF subunits, importantly, some of these studies have analysed HIF subunit binding partners and post-translational modifications under normal oxygen tension and following hypoxia.

Since the discovery of these important oxygen-sensitive transcription factors and enzymes, the use of ‘omic approaches such as transcriptomics and genomics has greatly propelled the field's knowledge, importance and reach into other disciplines. From the original physiology to molecular and cellular biology, medicine, veterinary sciences, chemistry and drug discovery, the impact of hypoxia research is now clear in all these areas. More recently, proteomics, epitranscriptomics and metabolomics have also started to be used in the field of hypoxia. This review will focus on how the use of omics and genome wide technologies where employed, has led to seminal discoveries in the research area of hypoxia (Figure 1).

## The use of transcriptomics techniques in hypoxia

Fluctuations in oxygen availability/demand initiate co-ordinated responses, involving changes in the regulation of gene expression, protein stability and function, which impinge on many aspects of physiology and cell biology [14]. Central to response and adaption to reduced oxygen tensions is the activation of gene transcriptional changes, primarily mediated by HIFs [15–17]. Additional transcription factors also play a role in co-ordinating transcriptional responses to hypoxia [18], these include NF-κB [19], REST [20], p53 [21] and MYC [22]. While initial investigation into gene expression changes in hypoxia was limited to individual or small groups of genes, high throughput transcriptomic profiling technologies have enabled researchers to measure changes in gene expression across the transcriptome. Initial work using microarrays and transcription start site (TSS)-sequencing (seq), and later work using RNA-seq, has the led to the identification of more than 2000 hypoxia responsive genes. Collectively, these data demonstrate cell/tissue specific transcriptome responses, with an underlying core hypoxia responsive gene signature [23–27]. Not surprisingly, these genes are part of hypoxia responsive pathways including metabolism, apoptosis, angiogenesis and chromatin regulation. While there is a degree of conservation of transcript up-regulation in response to hypoxia across cell types, down-regulated transcripts are very poorly conserved across cell types. The importance of gene transcriptional repression and molecular mechanisms behind them is less well understood than that of transcriptional activation [28]. Further to regulation of protein coding gene expression, hypoxia triggers changes in expression of microRNAs and other non-coding RNAs [29,30]. These discoveries have been driven through the use microRNA arrays [31–34], RNA-seq [35] and global run-on (GRO)-seq [36]. With decreasing costs and turnaround times for transcriptomics, it is now more feasible for research groups to conduct large-scale transcriptome profiling experiments. This is exemplified by recent work analysing the transcriptome in response to hypoxia across 31 different breast cancer cell lines [37]. Transcriptomics has also been a powerful tool in characterising the dependence of HIF and its expanding list of co-regulators, on gene transcriptional changes in hypoxia. The CDK8 mediator complex [38], KAT5 [39] and SET1B [40] are co-activators of HIF which are required for full transcriptional responses to low oxygen. REST is a key mediator of gene repression in hypoxia [41] and the histone deacetylase SIN3A is required for both full gene transcriptional repression, and activation in hypoxia [42].

A limitation of microarrays and RNA sequencing is they only provide a measure of steady state RNA levels without any information on the relative contributions of RNA transcription and decay rates. Methods detecting nascent RNA, namely metabolic RNA pulse labelling sequencing, GRO-seq and precision run-on (PRO)-seq, have elucidated direct transcriptional changes in response to low oxygen [36,38,42–45], and determined the relative contributions of transcription and decay to changes in mRNA levels [44]. These data find that transcription is the major contributor to changes in transcript levels in response to hypoxia and that these changes are mainly HIF dependent.

Most of our knowledge of transcriptome responses to varying oxygen levels comes from bulk cell analysis and despite the recent advances in single cell RNA sequencing, single cell resolution of transcriptome changes in response to oxygen fluctuations is lacking. The effects of chronic hypoxia on the transcriptome of single cells in the retina [46], and intermittent hypoxia on the transcriptome of single cells in the lung [47] of mice have been identified. However, there are currently no published studies using single cell RNA-seq in response to acute hypoxia, perhaps due to technical challenges in avoiding reoxygenation. Indeed, improvements and adaptations of protocol to sample preparation will allow single cell technologies to be used in hypoxia research in the future. While transcriptomics has no doubt been important in improving our understanding of gene regulation in hypoxia, some of the major mechanistic insights in this area have come through genomics approaches, often integrated with transcriptomics. These are discussed below.

## Genomic approaches used in hypoxia research

Genomics tools have been fundamental to understanding gene transcription and chromatin regulation in hypoxia. This includes characterising HIF direct and indirect transcriptional control, identifying the chromatin environment as determining cell type specificity of hypoxia/HIF responses, defining the distinct binding profiles and transcriptional outputs of HIF-1α and HIF-2α, and the discovering new cellular oxygen sensors.

Chromatin immunoprecipitation (ChIP)-chip microarrays [48–50] and ChIP-seq [35,40,42,45,51–58] have identified thousands of HIF binding sites across the genome, with HIF binding at hypoxia response element (HRE) at promoter proximal and distal regions. These analyses demonstrate cell type variations in HIF binding profiles. Integrative analysis with transcriptomics has helped uncouple primary and downstream transcriptional cascades in response to hypoxia and delineate direct and indirect HIF regulated genes [15,17], with most gene repression in hypoxia being indirect of HIF [28]. Multiomics approaches have also shown HIFs typically transactivate genes through release of paused RNA pol II [35,38,45]. Additionally, HIF binding to HREs is determined by the local chromatin environment, with HIFs binding preferentially to accessible chromatin, pre-loaded with RNA pol II [15–17]. Interestingly, despite sharing the same HRE sequence, HIF-1α and HIF-2α subunits have mainly distinct transcriptional regulatory outputs and genomic binding patterns which, together with cell specific isoform expression and regulation, contribute to their distinct functional outputs [15,52,55,58]. HIF-1α containing dimers typically have a preference for gene proximal HREs, whilst HIF-2α typically has a preference for gene distal HREs. Why these preferences have arisen is still unclear. HIF-3α is the least well studied of the three HIF-α isoforms, due to its many transcript variants, and complex tissue specific expression patterns [59]. One study to date has used ChIP-seq to map genome-binding sites of an overexpressed HIF-3α variant [60] finding significant differences compared with the other HIFs. Multiomics approaches will help define HIF-3α variant signalling and functions.

Given the drastic changes in gene expression in hypoxia, it is perhaps not surprising that the chromatin environment is also sensitive to oxygen. Post-translational modifications (PTMs) on histones and DNA, including histone methylation, histone acetylation and DNA methylation are associated with transcriptional regulatory mechanisms. ChIP-seq studies have shown hypoxia induces redistribution of the histone methylation landscape co-ordinating changes in hypoxia gene expression [40,61–64]. This work has contributed to the identification of specific JmjC histone demethylases, namely lysine demethylase 6A (KDM6A) and KDM5A, as oxygen sensors [62,64], determining the dependence of SET1B on H3K4me3 and gene expression changes in hypoxia [40], and elucidating the role of KDM4B and KDM6B in regulating hypoxic VEGFA expression and angiogenesis [43]. Furthermore, genome wide mapping of H3K27ac, a histone modification associated with transcriptionally active/poised genes, finds hypoxia induces changes in H3K27ac at gene loci correlating with effects of hypoxia on gene expression [42].

Analysis of the DNA methylome, using DNA immunoprecipitation sequencing (DIP)-seq, reveals DNA hypermethylation in hypoxia [65]. Mechanistically this occurs via ten eleven translocase (TET) enzyme oxygen sensing, with their involvement in DNA demethylation inhibited in low oxygen [65]. Multi omics incorporating DIP-seq in hypoxia has also shown that DNA methylation repels HIF subunit binding to HREs, with DNA methylation patterns helping define cell type specific responses to hypoxia [66].

Genomics approaches have also been used to investigate chromatin organisation and oxygen sensing. Most HREs overlap with DNase hypersensitivity sites which are present in normal oxygen tensions, indicating hypoxia is not required to open chromatin for HIF binding [35]. Assay for transposase-accessible chromatin (ATAC)-seq analysis has found hypoxia induces dynamic changes in chromatin accessibility in cell culture models [67–70]. The roles of HIF and 2-OGDD oxygen sensing in hypoxia-induced chromatin accessibility changes are, however, currently unknown and require further investigation. Chromosome conformation capture techniques enable analysis of interactions between genomic loci. Capture-C of a panel of HIF promoter proximal binding sites finds that that HIF promoter distal binding occurs at pre-established and primed, promoter enhancer loops [57]. Pertaining to genome wide topologically associated domains, Hi–C in endothelial cells identified a few long range interactions in that are acquired or lost in response to hypoxia [71]. The acquired sites were linked to hypoxia transcriptionally up-regulated genes [71].

Technical limitations with ChIP-seq makes obtaining high quality genome wide occupancy data for transient and/or low abundance chromatin binding factors difficult and expensive. Newer genome occupancy sequencing technologies, namely cut and run, and cut and tag, are tackling these limitations. Use of these new technologies should facilitate genome wide mapping of chromatin/transcription regulators in response to oxygen fluctuations, such as chromatin remodellers, which is likely currently lacking due to prior technical limitations. Chromatin profiling approaches to study oxygen sensing have thus far been limited to bulk cells. However, with advances in single cell chromatin profiling approaches, researchers can now start to investigate chromatin and oxygen sensing at the single cell level. The challenge for future studies is to elucidate the complex cross-talk between the chromatin environment, gene transcription and oxygen signalling. Multiomics approaches will no doubt take centre stage in making progress in this area.

## Defining the oxygen dependent proteome by mass spectrometry

The critical nature of the HIF-dependent changes in gene expression in response to hypoxia has led to a focus on identification of oxygen-dependent changes in transcription and chromatin architecture [18,72]. However, hypoxia's impact on protein turnover, gene-specific and global mRNA translation, all affect the composition of the proteome [73,74]. Systematically measuring changes in protein abundance by quantitative proteomic mass spectrometry (MS) analysis is the preferred approach to understand the complexity of gene expression under hypoxic stress at the protein level [75,76]. There have been several studies using metabolic or chemical labelling of complex protein samples to allow quantification in complex biological samples derived from cells cultured at different oxygen tensions [77–80]. Important new hypoxia targets have been validated following these unbiased screens, including a cross-talk between G protein-coupled receptor (GPCR) signalling to prevent apoptosis in hypoxic cancer cells [80], down-regulation of mitochondrial ribosome protein levels in hypoxic cervical cancer cells [77] and identification of LOX as an important mediator of metastasis in breast cancer [79]. It should, however, be noted that both GPRC5A and LOX are induced at the mRNA level in response to hypoxia in a HIF-dependent manner and could have been identified through the transcriptomic approaches outlined above [79,80]. Despite these advances, identifying hypoxia-induced proteins analysis of pooled populations of cells is challenging. Many key signalling events may only be happening in only a key subset of the cellular population (e.g. due to mixed cell types, position in the cell cycle, localised nutrient availability), which will be key in understanding the role of hypoxic signalling in the context of diseases such as cancers that contain heterogenous cell populations. The development and application of single cell proteomic approaches will allow the identification and characterisation of subcellular populations to understand the role of hypoxic signalling on an individual cell basis and may reveal key drivers and key cell types driving hypoxia signalling in a mixed cell population [81].

The real power of proteomics experiments in the context of large groups of cells may be to understand the role of hypoxia in changing the total proteome through subtle changes to many genes through modulating global translation rates [73,76]. Indeed, the primary observations from pulse chase stable isotope labelling with amino acids in cell culture (SILAC) experiments is the rapid and profound suppression of *de novo* protein synthesis, and these types of experiments will help to understand how acute hypoxia alters the proteome of hypoxic cells [73]. Coupling experimental data with pathway analysis of these global changes to the cellular proteome will enhance our understanding how cells ‘tune’ their proteome to restore oxygen homeostasis following hypoxic stress.

## Advances in understanding HIF signalling by mass spectrometry

In addition to providing valuable insight into hypoxia-induced protein targets, unbiased MS experiments have been critical in identifying the key mediators of the hypoxic response by defining changes in protein–protein interactions and PTMs of HIF transcription factors [82,83]. The activity of HIF, although primarily controlled by the activity and availability of the HIF-α subunits by PHDs and VHL, can be modulated and tuned by other modifying enzymes and interacting proteins [3–5,84]. Unbiased MS experiments have revealed that the PTM landscape and interaction partners if HIF transcription factors are in fact extremely complex [82,85]. The phosphosite plus repository of post-translational modifications of individual proteins either identified from MS or low throughput approaches, reveals that HIF-1α, HIF-2α, HIF-3α and HIF-1β have 73, 39, 20 and 29 unique PTMs, respectively [86,87]. The number of these modifications, ranging from phosphorylation, acetylation, ubiquitylation, methylation and acetylation of amino acids, and the variety of ways they can be deposited on the HIF subunits, confers a great deal of potential combinatorial complexity to control HIF activity. However, this is likely to be an underestimation of the real level of PTMs present. Recent detailed analysis specifically analysing phosphorylation of HIF-1α and HIF-2α identified 41 and 39 different phosphosites on HIF-1α and HIF-2α respectively, including evidence of non-canonical phosphorylation of cystine residues [85]. Critically this study examined changes to the phosphorylation landscape on HIF-1α and HIF-2α in response to both normal oxygen and hypoxic conditions in cervical carcinoma cells [85]. Interestingly, more than 50% of the mapped phosphorylation sites on the HIF-α subunits were differentially observed at different oxygen tensions [85]. Further analysis may reveal that the dynamic nature of this combinatory PTM code of HIF can alter its activity in response to specific stimuli or timing, as seen in the combinatorial modification of the NF-κB transcription factor in response to different stimuli [88].

PTMs of HIF transcription factors can control protein stability, interaction with DNA or mediate protein–protein interactions with several co-activator and co-repressor proteins [82,89]. Some of these, such as the interaction between HIF-1α and HIF-2α, with their co-activator p300/CBP, were identified in candidate-based approaches, but many of the key interaction partners have been identified using unbiased systematic screens [82,85]. Early studies to find interacting proteins of HIF-1α and HIF-2α used SILAC-based proteomics to identify 44 proteins that interacted with HIF-1α TAD, 42 that interacted with the HIF-2α TAD and 146 that interacted with both [90]. In this analysis the JmjC histone demethylase, KDM4C, was characterised as a key HIF-1α interactor and shown to be required for HIF-1-dependent metastasis [90]. A recent comprehensive study of HIF-1α and HIF-2α full length has revealed a much larger number of HIF interactors [85]. Intriguingly, this study found most of the common interactors to be oxygen-independent and that the oxygen-dependent interactions were very specific to either HIF-α isoforms [85]. Although, no further validation of these targets was performed, the disparity between the oxygen induced HIF-1α and HIF-2α interactors is intriguing due to the sometime distinct functions of HIF-1α and HIF-2α containing heterodimers. Additional work, using different modes of data acquisition for the instruments such as switch from data dependent to data independent mode, might lead to a more comprehensive analysis of HIF-α interacting partners.

## Proteome wide PTM analysis in hypoxia using mass spectrometry

Like most other signalling pathways, hypoxia is predicted to change the PTM landscape for the cell's proteome. Given, that hydroxylation and methylation levels are under direct control of dioxygenases, theses PTMs should be significantly altered in cells experiencing hypoxia. Unfortunately, studies investigating protein methylation have not been reported outside of histones [62,91]. Several studies have investigated replace with hydroxylation, not necessarily at the proteome level but using more directed approaches such as altering PHDs or FIH levels [92–98]. However, methodological improvements in sample preparation are needed to truly address hypoxia-modulated hydroxylation at the proteome wide level. This is an area that is still quite controversial in the field of hypoxia research [97].

Sumoylation is the covalent conjugation of the ubiquitin-like protein called sumo to another protein [99,100]. Sumo is present in three different forms, Sumo-1, Sumo-2 and Sumo-3 [99,100]. Conjugation of sumo to a protein can result in a variety of functional changes, from changes in cellular localisation, to change in the interaction partners or enzymatic function [99,100]. Sumo has been shown to modulate HIF-1α function in a variety of ways [101]. However, studies using sumoylation analysis in hypoxia using MS have shown that sumoylation is broader than just HIF and help co-ordinate the cellular response needed for hypoxia [101–105]. Furthermore, this has led to the realisation that the enzymes that remove sumo from proteins, called sumo proteases, SENPs, are inhibited in hypoxia, by a yet unknown mechanism [106].

Phosphorylation is a crucial PTM regulating a many of the intra- and intercellular cell signalling pathways induced in normal and malignant hypoxic cells. As discussed, changes in phosphorylation states play key roles in regulating HIF signalling; however, large-scale changes in the phosphoproteome are observed in cells exposed to hypoxic stress [107,108]. A recent phosphoproteomic study analysing the hypoxia-induced phosphoproteome from four distinct melanoma cell lines revealed both a core set of phosphosites induced by hypoxia, and a subset of cell line specific phosphorylation events [108]. This analysis revealed that the mitogen-activated protein kinases (ERK1 and ERK2) and casein kinase were predicted to be active, with phosphorylation sites on the kinases differentially phosphorylated, and hypoxia-induced hyperphosphorylation of several predicted substrates [108]. These results indicate that hypoxia-induced signalling pathways are not only mediated by the PHD/VHL/HIF pathway. Incorporation of data sets derived from multiple cell types, both normal and malignant, would define cell type specific phosphorylation events and which are critical across all human cells.

## Metabolomics and hypoxia research

One major biological process altered by changes to oxygen availability is cellular metabolism [14]. Furthermore, oxygen dependent dioxygenases such as PHDs and other enzymes belonging to this class, require 2-oxoglutarate for their enzymatic activity [11,12]. As such, it is important to understand how changes in oxygen leads to changes in metabolites in the cell, that can give rise to dramatic alterations in cellular processes. While targeted approaches using more traditional methods revealed changes to some metabolites such as lactate, several studies have reported the use of metabolomics to determine changes in such molecules in an unbiased manner.

Unbiased metabolomics can be achieved via nuclear magnetic resonance (NMR) or using MS [109]. Advances in instrumentation and standard in methodology have allowed for metabolomics to become more commonly used [110]. However, only a few studies have been directed at the hypoxia response.

Work using metabolomics, mostly investigating diseased states, have helped to the identification of specific changes elicited by hypoxic stress [111–114]. These include changes to levels of succinate [115], fumarate [116,117], various amino acids [118,119] and even identification of oncometabolites such as 2-hydroxyglutarate [120]. Despite confirmation of altered metabolism in cells exposed to hypoxia, all these studies have also highlighted tissue and cell specific responses, depending on normal function or additional genetic lesions present in the case studied. Since metabolism is inherently linked to all aspects of the cell's environment, vast changes in metabolomic profile can occur with a simple change in culture condition. Furthermore, the situation is even more complex when using human samples, where external factors such as diet, smoking and working environment can make significant alteration to the metabolome of an individual. Although metabolomics technologies have improved, its use in the area of low oxygen sensing is still behind approaches such as genomics and even proteomics [121]. The complex nature of the data obtained, and data analysis coupled with inherent sample variability and hence cost of the experimentation, has prevented this approach from being more widely used. However, is clear that more work using metabolomics will be complementary to other omics approaches already used in hypoxia research, such as transcriptomics and genomics. Hopefully, and like with the use of proteomics, improvements in sample preparation and analysis methods will make the use of metabolomics more readily accessible to researchers in the field of hypoxia.

## Epitranscriptomics, an emerging area for hypoxia research

RNA epigenetics (epitranscriptomics) is a rapidly growing and exciting field that represents an additional layer in the control of genetic information. RNAs carry a diverse range of chemical modifications; one of the most abundant in eukaryotes is N6 methyladenosine (m6A). Modulation of this highly dynamic and reversible modification on internal regions of mRNA and other types of nuclear RNA is orchestrated by m6A writers and erasers, with readers of the modification important in conferring its functional output [122]. m6A impinges on all aspects of RNA biology and is an important regulator of various biological processes [123,124]. Initial investigations into the role of m6A in oxygen sensing reveals changes in total m6A cells in response to hypoxia [125] and elevated m6A levels at a subset of hypoxia inducible genes, which increases transcript stability and translation [126]. In addition, hypoxia induced, HIF-dependent removal of *NANOG* mRNA via ALKBH5 activates a breast cancer cell phenotype [127]. m6A enrichment sequencing or direct RNA sequencing approaches enable identification and quantification of m6A across the transcriptome. Several m6A transcriptome screens have identified m6A methylation sites on HIF-1α [128–133] and m6A modification on HIF-1α has recently been confirmed by RNA ImmunoPrecipitation-PCR analysis [134]. To date only one study has mapped the m6A epitranscriptomic is response to low oxygen [135]. This study reveals hypoxia-induced changes in m6A at over 2000 transcripts. Multiomics analysis with RNA-seq and proteomics shows these changes are enriched at hypoxia responsive genes/proteins and researchers demonstrate that the RNA demethylase ALKBH5 is required for hypoxia-induced m6A demethylation [135]. Reprogramming of the epitranscriptome in response to oxygen fluctuations may, therefore, be crucial for shaping gene expression changes and is an important new area for the field.

## Genome wide genetic screens in hypoxia

Although not necessarily an omics approach, genome wide screen using gene targeting approaches such as siRNAs and CRISPR have also contributed to our understanding of hypoxia. These approaches and their impact in the field have been nicely reviewed [136] and have recently led to the identification of SET1B as a specificity factor for HIF-dependent gene induction in hypoxia [40], defined mitochondrial genes, essential for the viability of tumour cells in hypoxia [137], used in synthetic lethality for hypoxic tumours [138]and identified susceptibilities in hypoxic liver cancer [139,140]. It is thus clear, that genome wide screens can still contribute much needed knowledge to this area.

## Final thoughts

The ability to conduct unbiased, omics analysis has greatly propelled the hypoxia research field forward. Perhaps, not surprisingly, sequencing related technologies have the front stage in this revolution. However, with technology development in the MS instrumentation, other approaches such as proteomics and metabolomics will become more accessible to the community and used more routinely in the future. However, bioinformatic expertise in these areas is still difficult and despite the advances in methods and instruments, data analysis, interpretation and integration remain a barrier for a research area dominated by physiologists, cell biologists and biochemists. As such, a team approach is much needed to bring bioinformatics knowhow to hypoxia research. Moving forward, systems and mathematical based approaches integrating omics datasets in a statistically rigorous fashion will allow us to comprehensively and accurately model the cellular response to hypoxia.

## Open Access

Open access for this article was enabled by the participation of University of Liverpool in an all-inclusive *Read & Publish* pilot with Portland Press and the Biochemical Society under a transformative agreement with JISC.

## Abbreviations

2-OGDD: 2-oxoglutarate dependent dioxygenase
CBP: CREB binding protein
ChIP: chromatin immunoprecipitation
DIP: DNA immunoprecipitation sequencing
FIH: factor inhibiting HIF
GRO: global run-on
HIFs: hypoxia inducible factors
HRE: hypoxia response element
LOX: lysyl oxidase
MS: mass spectrometry
PHDs: prolyl-hydroxylases
PRO: precision run-on
PTMs: post-translational modifications
SILAC: stable isotope labelling with amino acids in cell culture
VHL: Von Hippel–Lindau Tumour Suppressor
